# Value of catecholamine levels in the differential diagnosis of vasovagal syncope and psychogenic pseudosyncope in children

**DOI:** 10.3389/fped.2024.1281196

**Published:** 2024-05-31

**Authors:** Hua Wang, Wandong Ma, Mei Jin, Bo Li, Suzhen Sun

**Affiliations:** ^1^ Department of Pediatrics, Hebei Medical University, Shijiazhuang, China; ^2^ Department of Pediatric Cardiology, Children’s Hospital of Hebei Province, Shijiazhuang, China; ^3^ Hebei Provincial Key Laboratory of Pediatric Cardiovascular Disease, Shijiazhuang, China; ^4^ Department of Neurosurgery, Hebei General Hospital, Shijiazhuang, China; ^5^ Department of Pediatric Neurology, Children’s Hospital of Hebei Province, Shijiazhuang, China

**Keywords:** vasovagal syncope, psychogenic pseudosyncope, catecholamine, differential diagnosis, head-up tilt tests

## Abstract

**Background and purpose:**

Vasovagal syncope (VVS) and psychogenic pseudosyncope (PPS) can be difficult to distinguish, given their similar clinical presentations. This study was conducted to explore the clinical value of catecholamine levels in the differential diagnosis of VVS and PPS in children.

**Methods:**

This retrospective case-control study was conducted with data from children with VVS and PPS who underwent head-up tilt tests (HUTTs) at the Children's Hospital of Hebei Province between March 2021 and March 2023. The data collected were baseline clinical characteristics, HUTT results, serum catecholamine levels in the supine and upright positions, and 24 h urinary catecholamine concentrations. These variables were compared between the VVS and PPS groups.

**Results:**

From 328 potentially eligible cases, 54 (16.46%) cases of VVS and 24 (7.32%) cases of PPS were included in the analysis. No significant difference in age, sex, body mass index, or syncope frequency was observed between the VVS and PPS groups. The main predisposing factors for syncope were body position changes in the VSS group (83.33%) and emotional changes in the PPS group (41.67%). The episode duration was significantly shorter in the VSS group than in the PPS group (4.01 ± 1.20 vs. 24.06 ± 5.56 min, *p* < 0.05). The recovery time was also shorter in the VVS group than in the PPS group (1.91 ± 0.85 vs. 8.62 ± 2.55 min, *p* < 0.05). Relative to patients with PPS, those with VVS had significantly higher serum epinephrine (EP) levels in the upright position [199.35 (102.88, 575.00) vs. 147.40 (103.55, 227.25), *p* < 0.05] and lower serum epinephrine levels in the supine position [72.70 (42.92, 122.85) vs. 114.50 (66.57, 227.50), *p* < 0.05].

**Conclusions:**

Serum EP levels have potential value in the differential diagnosis of VVS and PPS.

## Introduction

Transient loss of consciousness (TLOC) is a common clinical symptom that accounts for approximately 3% of all emergency department visits (1, 2). It can be caused by mechanisms ranging from reflex syncope to arrhythmia and heart block (3). Syncope, the most common cause of TLOC, is characterized by the inability to maintain an autonomous body position due to a reduction in oxygen delivery to the central nervous system induced by cerebral hypoperfusion. It is characterized by sudden TLOC followed by rapid and complete recovery (4). Vasovagal syncope (VVS) accounts for approximately 60%–70% of syncope cases and is especially common among children and adolescents (5). It is an abnormal response mediated by the autonomic nervous system and can be divided into vascular inhibitory, cardiac inhibitory, and mixed types (6). Psychogenic pseudosyncope (PPS) is another clinical syndrome that occurs without defective cerebral perfusion or function (7). As PPS and VVS share clinical manifestations such as falling and recurrent TLOC episodes, their timely and accurate diagnosis in symptomatic children is difficult (8, 9). It is essential, however, as the treatment and prognosis of these two conditions are quite different.

The head-up tilt test (HUTT) is a routine clinical test used in the differential diagnosis of VVS and PPS, but it alone is not sufficient due to its low sensitivity (10). In addition, the HUTT is time consuming and inconvenient and may induce shock, limiting its broad clinical application. Thus, the development of simpler, more specific and reliable methods to distinguish VVS from PPS in clinical scenarios is needed. Several groups are currently trying to find innovative methods to aid the differentiation of VVS and PPS in children (10, 11).

Catecholamines, including epinephrine (EP), norepinephrine (NE), and dopamine (DP), are important neurotransmitters secreted from the adrenal medulla. Extensive neurohumoral changes are related to VVS onset (12). As early as 1965, Chosy and Graham (13) reported that the urine EP level was higher in patients with than in those without VVS. Subsequently, changes in catecholamine levels associated with the pathogenesis of VVS have been foci of research. In the upright position, the NE and EP levels rise to a greater extent in patients with than in those without syncope. In proximity to syncope, however, the EP level continues to rise, peaking at the end of episode, while the NE level returns to normal (14–16). Based on HUTT results, EP has been identified as a possible contributor to VVS susceptibility (17). However, the potential syncope-related diagnostic value and mechanisms of action of catecholamines remain unclear. This study was conducted to evaluate whether catecholamine levels can be used as auxiliary indicators for the differential diagnosis of VVS and PPS in clinical practice.

## Method

### Participants

In this retrospective case-control study, children with unexplained syncope who presented to the Children's Hospital of Hebei Province between March 2021 and March 2023 were considered for inclusion in this study. The inclusion criteria were: (1) diagnosis of VVS or PPS, (2) age <18 years, (3) HUTT performance, (4) measurement of supine and upright plasma catecholamine levels, and (5) measurement of 24-hour urine catecholamine concentrations. Patients (1) diagnosed with PPS and VVS and those with (2) syncope caused by cardiogenic, neurogenic, and other diseases and (3) insufficient clinical information were excluded. The hospital's ethics committee approved the study (Medical Ethics no. 24), the patients’ [legal guardian/next of kin] provided written informed consent to participate in this study. The clinical data were collected from the electronic medical records system of our hospital.

### Diagnoses

VVS was diagnosed in accordance with the 2018 guidelines for the diagnosis and treatment of syncope in children and adolescents in China (18). The criteria were: (1) a clear history of syncope with spontaneous recovery; (2) attacks usually induced by prolonged uprightness, mental stress, and environment factors, such as sultry; (3) HUTT positivity; (4) sudden hypotension and/or inappropriate bradycardia during onset; and (5) the exclusion of other disorders, such as cerebrovascular, cardiogenic, and metabolic diseases.

PPS was diagnosed according to the fifth edition of the Diagnostic and Statistical Manual of Mental Disorders (19). The criteria were: (1) a clear history of recurrent syncope with spontaneous recovery; (2) eye closure and muscle tone loss; (3) normal heart rate (HR) and blood pressure (BP) before, during, and after the clinical event; and (4) the exclusion of other disorders, such as neurogenic, cardiogenic, and metabolic diseases (18).

### HUTT performance and catecholamine detection

The HUTT was performed according to the 2018 guidelines for the diagnosis and treatment of syncope in children and adolescents in China (18). The subjects were asked to fast for at least 4 h before the test and to stop any vasoactive medication for at least five half-lives. The test was conducted in a temperature-controlled, quiet, dimly lit room. After 10 min rest, the subjects’ HR, BP, and heart function were recorded continuously with an ambulatory BP meter, an echocardiographic monitor, and a tilting bed (MedStandard, Suzhou, China) in the supine position and then with a head-up (60°) tilt for 45 min or until a positive response occurred. Blood samples in the supine position were collected firstly. And after 10 min in the upright positions, the next sample for upright positions were collected. In addition, 24 h urine samples were collected. The levels of catecholamines in these samples were detected by high-performance liquid chromatography/mass spectrometry (Sigma Aldrich, St. Louis, MO, USA) according to the manufacturer’s instructions.

### Data collection from medical records

Participants’ basic clinical and demographic data, including sex, age, and body mass index (BMI), were retrieved from their medical records. Data on their syncope-related past medical histories, such as the LOC duration, attack frequency, predisposing factors, and family history, were characterized via self-administered questionnaire. A dedicated staff member recorded the medical information, and another investigator independently checked it.

### Statistical analysis

The statistical analyses were performed using SPSS (version 24.0; IBM Corporation, Armonk, NY, USA). The normality of data distribution was assessed using the Shapiro–Wilk test. Normally distributed measurement data are expressed as means ± standard deviations and were compared between groups using the unpaired *t* test. Non-normally distributed variables are expressed as medians with interquartile ranges and were compared between groups using the nonparametric Mann–Whitney *U* test. Enumeration data are expressed as rates or percentages and were analyzed using the chi-squared test. Differences were considered to be significant with *p* < 0.05.

## Results

### Baseline characteristics

**Figure 1:**
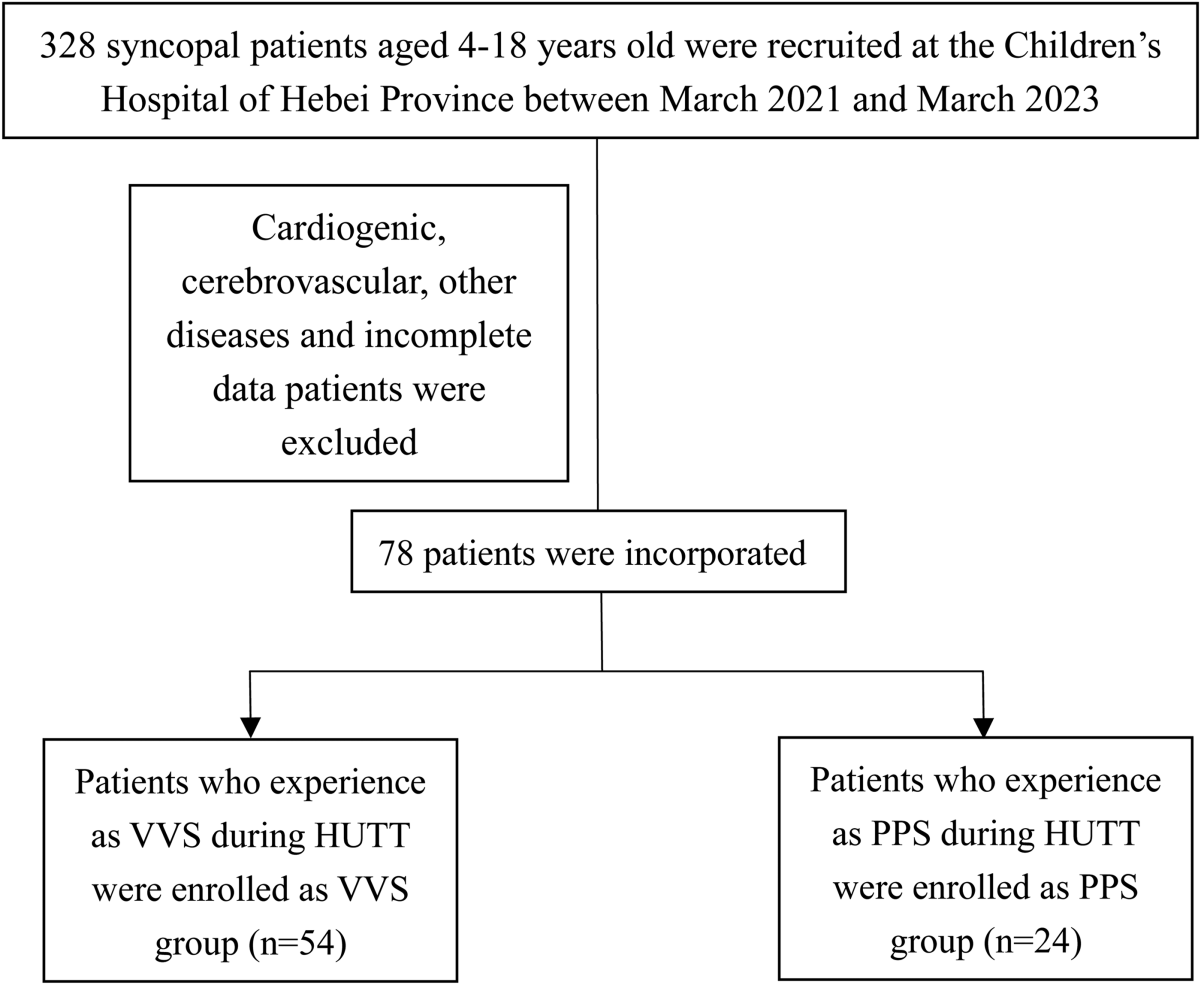
Flow of patient inclusion.

Of 328 patients with syncope assessed at the hospital during the study period, including HUTT performance 182 (55.48%) and 24 (7.33%) patients were diagnosed with VVS and PPS, respectively. After the exclusion of patients for whom catecholamine measurements were lacking, 54 patients with VVS (21 males, 33 females) and 24 patients with PPS (11 males, 13 females) were enrolled in the study (Figure 1). The patients’ baseline and demographic characteristics are provided in Table 1. No significant difference in age, sex, or BMI was observed between groups.

**Table 1:** Clinical characteristics of patients with VVS and PPS.

	VVS (*n* = 54)	PPS (*n* = 24)	*P*-value
Causative factors exposed			
Posture (yes/no)	45 (83.33%)	6 (25.00%)	0.00
Emotional (yes/no)	3 (5.56%)	10 (41.67%)	0.00
General information			0.56
Sex			
Male	21 (38.89%)	11 (45.83%)	
Female	33 (61.11%)	13 (54.17%)	
Age (years)	11.26 ± 2.49	10.63 ± 2.65	0.31
BMI (kg/m^2^)	17.97 ± 3.08	18.6 ± 1.97	0.36
History duration (month)	2.00 (1.00,12.00)	1.00 (1.00,2.00)	0.00
Frequency of syncope (time)	3.00 ± 0.31	3.61 ± 0.43	0.60
Syncope duration (min)	4.01 ± 1.20	24.06 ± 5.56	0.00

Data are presented as the mean ± standard deviation, median (interquartile range), or *n* (%).

VVS, vasovagal syncope; PPS, psychogenic pseudosyncope; BMI, body mass index.

### Clinical features

The main predisposing factors for syncope were body position changes in the VSS group (83.33%) and emotional changes in the PPS group (41.67%; *p* < 0.05; Table 1). Syncope history durations were longer in the VSS group than in the PPS group [2.00 (1.00, 12.00) vs. 1.00 (1.00, 2.00) months, *p *< 0.05; Table 1]. The frequency of syncope did not differ significantly between the VVS and PPS groups (3.00 ± 0.31 and 3.61 ± 0.43 events, *p* = 0.6; Table 1). The syncopal episode duration was significantly shorter in the VVS group than in the PPS group (4.01 ± 1.20 vs. 24.06 ± 5.56 min, *p* < 0.05; Table 1). HUTT results indicated that the recovery time was shorter in the VVS group than in the PPS group (1.91 ± 0.85 vs. 8.62 ± 2.55 min, *p* < 0.05; Table 2). No significant difference in the baseline HR, systolic or diastolic BP, or positive response time was observed between groups (*p* > 0.05).

**Table 2:** HUTT data from patients with VVS and PPS.

	VVS (*n* = 54)	PPS (*n* = 24)	*P*-value
HUTT data			
Basic HR (bpm)	75.74 ± 11.67	79.96 ± 11.33	0.14
Basic SBP (mmHg)	106.54 ± 13.49	107.08 ± 8.87	0.86
Basic DBP (mmHg)	65.21 ± 6.82	62.50 ± 7.44	0.71
Time of positive response (min)	13.00 (6.00, 27.00)	18.50 (12.00, 22.00)	0.64
Recovery time (min)	1.91 ± 0.85	8.62 ± 2.55	0.00

Data are presented as the mean ± standard deviation or median (interquartile range).

HUTT, head-up tilt test; VVS, vasovagal syncope; PPS, psychogenic pseudosyncope; HR, heart rate; SBP, systolic blood pressure; DBP, diastolic blood pressure.

### Catecholamine levels

We then performed catecholamine levels analysis among VVS and PPS patients. As presented in Table 3, the serum NE [upright: 1,195.00 (790.00, 1,982.63) pmol/L vs. 1,080.60 (737.00, 1,323.90) pmol/L, *p* > 0.05; supine: 72.7 839.00, (458.65, 1,074.00) pmol/L vs. 950.50 (555.00, 1,159.80) pmol/L, *p* > 0.05] and NP [upright: 35.25 (33.00, 43.13) pmol/L vs. 44.50 (33.00, 59.00) pmol/L, *p* > 0.05; supine: 34.00 (33.00, 43.00) pmol/L vs. 43.00 (33.00, 56.00) pmol/L, *p* > 0.05] levels did not differ between groups, regardless of the body position. Notably, relative to patients with VVS, those with PPS had significantly lower serum EP levels [199.35 (102.88, 575.00) vs. 147.40 (103.55, 227.25) pmol/L, *p* < 0.05] in the upright position and higher serum EP levels [72.7 (42.92, 122.85) pmol/L vs. 114.5 (66.57, 227.50) pmol/L, *p* < 0.05] in the supine position. As for 24-h urine sample, no significant difference was observed in EP [1,348.00 (1,043.25, 1,837.75) nmol/24 h vs. 1,260.00 (932.45, 1,551.00) nmol/24 h, *p* > 0.05], NE [77.30 (53.75, 105.63) nmol/24 h vs. 77.15 (58.85, 84.75) nmol/24 h, *p* > 0.05] and NP [15.75 (9.98, 28.20) nmol/24 h vs. 19.00 (14.48, 32.73) nmol/24 h, *p* > 0.05] between groups.

**Table 3:** Catecholamine levels in patients with VVS and PPS.

		VVS (*n* = 54)	PPS (*n* = 24)	*P*-value
The cat level of OH	EP	199.35 (102.88, 575.00)	147.40 (103.55, 227.25)	0.02
	NE	1,195.00 (790.00, 1,982.63)	1,080.60 (737.00, 1,323.90)	0.26
	DP	35.25 (33.00, 43.13)	44.50 (33.00, 59.00)	0.09
The cat level of Cl	EP	72.7 (42.92, 122.85)	114.50 (66.57, 227.50)	0.04
	NE	839.00 (458.65, 1,074.00)	950.50 (555.00, 1,159.80)	0.23
	DP	34.00 (33.00, 43.00)	43.00 (33.00, 56.00)	0.10
The cat level of 24 h U	EP	1,348.00 (1,043.25, 1,837.75)	1,260.00 (932.45, 1,551.00)	0.28
	NE	77.30 (53.75, 105.63)	77.15 (58.85, 84.75)	0.62
	DP	15.75 (9.98, 28.20)	19.00 (14.48, 32.73)	0.18

VVS, vasovagal syncope; PPS, psychogenic pseudosyncope; Cat, catecholamine; OH, orthostatism; Cl, clinostatism; EP, epinephrine; NE, norepinephrine; DP, dopamine; U, urine.

## Discussion

In the present study, we analyzed the clinical features, HUTT results, and catecholamine levels of children with VVS and PPS. Notably, we found that the serum EP level in upright posture and EP level in supine posture were statistical significance, suggesting that it can aid the differential diagnosis between VVS and PPS.

VVS is among the most common causes of syncope in children and adolescents and is triggered mainly by postural change and/or emotional stress (8, 20). It is characterized by sympathetic withdrawal and increased vagal tone (20). Most patients with VVS exhibit hypotension and bradycardia during attacks, which last for a few minutes and self-terminate (21). PPS is a TLOC entity, the prevalence of which may be underestimated in children (22, 23). It is believed to be a conversion (i.e., psychiatric) disorder. Generally, PPS attacks in children are induced by emotional stress, such as that caused by abuse, abandonment, or school phobia/transfer (9). This type of syncope usually occurs at rest, rather than during exertion. Presyncope symptoms may include dizziness, dyspnea with hyperventilation, and tingling (24). During a PPS episode, TLOC may last for several minutes or up to 50 min (22, 24). We initially assessed the demographic characteristics and clinical characteristics in VVS and PPS patients. Consistent with previous reports, no difference was observed in gender, age and BMI. Typical VVS was usually triggered by posture changes, while PPS was induced by emotional factors. Furthermore, syncope duration was significant longer in PPS patients when compared to VVS patients. Remarkably, patients with VVS regained complete consciousness within 1–2 min after syncope onset, whereas for PPS, the recovery time was approximately 8 min. A similar phenomenon was observed in another study (2, 11).

In clinical practice, physicians usually make initial differential diagnoses between these disorders based on clinical symptoms and HUTT results. In contrast to VVS, the typical feature of PPS is eye closure during an episode; another important difference is that the syncope duration is longer in PPS than in VVS. However, direct observation of entire episode courses rarely occurs in clinical settings. In this study, we also found that the syncope duration was longer in the PPS group than in the VVS group. Thus, a detailed information of disease during episode is helpful for disease diagnosis and management. However, it is not always possible to obtain such complete record in clinical settings. Indeed, evidence suggests that the number of PPS cases is grossly underestimated (11, 25). Consequently, finding a simple and reliable indicator for differential diagnosis between VVS and PPS is urgently needed.

Catecholamines, including EP, NE, and DP, are hormones play critical roles in regulating metabolism, immune function, BP, stress responses, and other essential biological processes (15, 26). However, whether the EP level can be used to distinguish VVS from PPS in children remains unclear. Herein, we found that the upright serum EP level was higher in patients with VVS than in those with PPS, whereas supine serum EP level was exactly the opposite. It suggests that the serum EP level in either upright or supine posture can serve as an auxiliary indicator for the differential diagnosis of PPS and VVS.

This study has some limitations. Due to the small sample, we could not determine the optimal cut-off value for the upright serum EP level for diagnosis. Thus, multi-center studies with large samples should be conducted in the future. Meanwhile, the practical application in clinic deserves further exploration.

## Conclusion

Our results suggest that the serum EP level can be used as an auxiliary indicator for the differential diagnosis of VVS and PPS, in combination with clinical symptoms and HUTT results.

## Data Availability

The raw data supporting the conclusions of this article will be made available by the authors, without undue reservation.
